# ONTraC characterizes spatially continuous variations of tissue microenvironment through niche trajectory analysis

**DOI:** 10.1186/s13059-025-03588-5

**Published:** 2025-05-08

**Authors:** Wen Wang, Shiwei Zheng, Sujung Crystal Shin, Joselyn Cristina Chávez-Fuentes, Guo-Cheng Yuan

**Affiliations:** Department of Genetics and Genomic Sciences, Icahn School of Medicine at Mount Sinai, New York, NY 10029 USA

## Abstract

Recent technological advances enable mapping of tissue spatial organization at single-cell resolution, but methods for analyzing spatially continuous microenvironments are still lacking. We introduce ONTraC, a graph neural network-based framework for constructing spatial trajectories at niche-level. Through benchmarking analyses using multiple simulated and real datasets, we show that ONTraC outperforms existing methods. ONTraC captures both normal anatomical structures and disease-associated tissue microenvironment changes. In addition, it identifies tissue microenvironment-dependent shifts in gene expression, regulatory network, and cell–cell interaction patterns. Taken together, ONTraC provides a useful framework for characterizing the structural and functional organization of tissue microenvironments.

**Supplementary Information:**

The online version contains supplementary material available at 10.1186/s13059-025-03588-5.

## Background

The behavior of a cell is not just determined by its intrinsic identity but also mediated through interactions with its external tissue microenvironment. Spatial transcriptomics offers an opportunity to dissect the spatial variations of the tissue microenvironment, which in turn can provide mechanistic insights into its role in mediating cell-state changes in development and diseases. However, it requires sophisticated computational methods to extract biologically relevant information from spatial transcriptomics data. While numerous methods have been developed to identify cellular neighborhoods [1–3] and spatial domains [4–12], these methods can only identify discrete spatial patterns.

Spatial trajectory analysis is an alternative approach that aims at modeling the spatially continuous variations. This is typically done by applying pseudotime analysis [13–17], which was originally developed for analyzing single-cell RNA-seq data, to the gene expression data and then map to the physical space. However, a major limitation of pseudotime analysis is that the trajectory is constructed on the basis of gene expression pattern similarities, whereas the spatial information is ignored. Recently, several methods have been developed to overcome this limitation by further incorporating spatial information. For example, stLearn constructs spatial trajectories by using a metric that combines the pseudotime analysis output and spatial distance [18], whereas SpaceFlow [7] and spatialPCA [19] modify the pseudotime analysis procedure by cell-state embedding using both spatial and gene expression information. While these extensions have led to improved performance, they cannot address the intrinsic conflict between cell-state and spatial continuity. As such, a new modeling framework is needed to fundamentally overcome this challenge.

Toward this end, we introduce **O**rdered **N**iche **Tra**jectory **C**onstruction (ONTraC for short) as a new modeling framework for spatial trajectory analysis. A key feature of ONTraC is that it treats a multi-cellular niche, rather than a single cell, as the basic unit. A niche trajectory is a spatially continuous path that connects niches with similar cell-type composition properties, which can also be viewed as a one-dimensional representation of the tissue microenvironment continuum. Unlike traditional trajectory analysis, ONTraC does not attempt to predict either temporal cell-state changes or lineage relationship but focuses on modeling the spatial landscape of tissue microenvironment changes independent of the cell lineage. As such, ONTraC provides a common framework for systematic investigation of the impact of tissue microenvironment variation.

Through extensive benchmark analyses, we show that ONTraC provides a unified framework that enables the systematic analysis and comparison of the impact of tissue microenvironment changes on mediating gene expression programs and facilitating cell–cell communications across multiple cell types. Multiple tutorials for showcasing the usage of ONTraC are available on the website (https://ontrac-website.readthedocs.io/).

## Results

### A modeling framework for niche trajectory analysis

ONTraC takes spatial location and cell-type annotation information as input and uses a niche as the basic unit of the tissue microenvironment. Here, we use the term “niche” to represent a multicellular, spatially localized region where different cell types may coexist and interact. In the remainder of the paper, a niche is computationally implemented as the multi-cellular region that contains the *k*-nearest neighbors of any cell, referred to as the anchoring cell of the niche, based on the spatial distance. The relationship between a cell and a niche is quantified by a cell-niche association score, whose value decreases with the distance from the anchoring cell. The overall property of a niche is quantified by a numerical vector that summarizes its cell type composition. While neighboring cells may have distinct cellular states, the values of the cell-type composition vectors are spatially continuous, provided that *k* is sufficiently large.

**Fig. 1:**
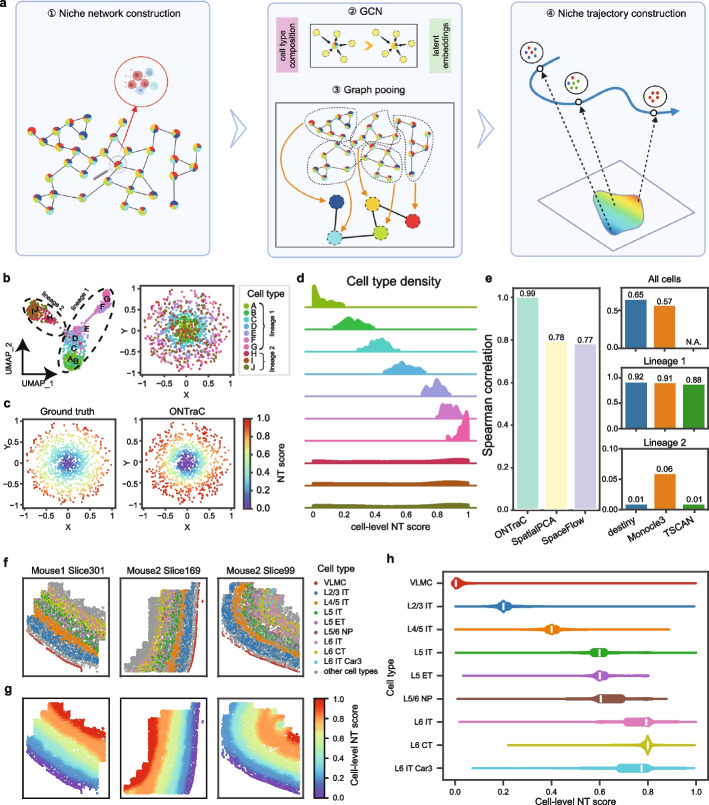
ONTraC captures spatially continuous changes in the tissue microenvironment. **a** A schematic of the ONTraC workflow. **b**–**e** Analysis of simulated_dataset_1. **b** UMAP (left) and spatial (right) distribution of cell types in simulated dataset 1. **c** Spatial distribution of ground truth (left) and ONTraC output (right) cell-level NT scores. **d** Cell type density along the reconstructed niche trajectory. **e** Bar plots showing the Spearman correlation between the ground truth and the ordering scores predicted by different methods. ONTraC, SpatialPCA, and SpaceFlow were applied only to the whole dataset, whereas the other methods were applied to the whole dataset, lineage 1, and lineage 2, respectively. **f**–**h** Analysis of the mouse motor cortex MERFISH data. **f** Spatial distribution of cell types on representative slices. **g** Spatial distribution of cell-level NT scores on representative slices. **h** Cell type density along the reconstructed niche trajectory

The main output of ONTraC is the niche trajectory (NT), which can be viewed as a one-dimensional representation of the tissue microenvironment continuum. As schematically shown in Fig. 1a and Additional file 1: Fig. S1, the ONTraC workflow for constructing NT includes the following steps. First, we construct a niche network connecting neighboring niches. The properties of each niche are summarized by its corresponding cell-type composition vector. Second, we construct a two-layer graph convolutional network (GCN) model [20] to encode input data into low-dimensional feature vectors while maintaining spatial continuity. Third, we adopt a graph pooling network with modified loss functions [21] to identify niche clusters and construct a niche cluster network based on their spatial distance. Fourth, we use the niche cluster network as the backbone to construct NT and determine the mapped location for each niche, which is called its NT score. Based on the cell-niche association scores, we also map each cell onto a specific location along the NT and refer to it as the cell-level NT score. The technical details are described in the Methods section.

To evaluate the performance of ONTraC, we applied it to four simulated datasets generated by superimposing various spatial patterns over a state-of-the-art single-cell RNA-seq data simulator [22]. As a proof-of-concept, we initially simulated an idealized dataset (simulated dataset 1, Additional file 2: Table S1, Additional file 3: Table S2), which contains the expression levels of 135 genes in 1000 cells sampled from 10 cell types, representing two cell lineages that were separated by a bifurcation point (Fig. 1b, left panel). While cells from lineage 1 are distributed with a circular pattern in progressive order, cells from lineage 2 are distributed randomly in space (Fig. 1b, right panel) (see Methods for more details). Applying ONTraC to simulated dataset 1 correctly recapitulated the spatial pattern of the niche organizations (Fig. 1c, d and Additional file 1: Fig. S2). The overall agreement between cell-level NT scores and the ground truth is very high (Fig. 1e, Spearman correlation = 0.99).

To test the robustness of ONTraC against parameter settings, we repeated the analysis by varying several key parameters, including running ONTraC with variable hyperparameters combinations. The results showed that ONTraC can yield stable results with different dimensionality of the latent space, the number of GCN layers, the *K* value for constructing niches, and the number of niche clusters. The results are highly reproducible (Additional file 1: Fig. S3).

We benchmarked the performance of ONTraC against three established pseudotime methods, including destiny [14], Monocle 3 [17], and TSCAN [23]. For each method, we mapped the derived pseudotime coordinates to the corresponding spatial locations and evaluated their performance using Spearman correlation. For destiny and Monocle 3, the Spearman correlation values were 0.65 and 0.57, respectively. Due to its treatment of branching events, in the TSCAN analysis a significant fraction (over 20%) of cells did not have any pseudotime values; therefore, the overall Spearman correlation could not be evaluated properly (Fig. 1e and Additional file 1: Fig. S2). Since pseudotime analysis is often carried out on subsets of cells along a common lineage, we further tested the performance of each pseudotime analysis method by using cells from each lineage separately. All three methods worked well for lineage 1 (Spearman correlation between 0.88 and 0.92) but performed poorly for lineage 2 (Spearman correlation between 0.01 and 0.06). This is expected because the spatial distribution of lineage 2 cells does not follow the lineage structure. This analysis suggests the performance of a pseudotime analysis method is highly sensitive to the presence of cells whose spatial distribution is uncorrelated with their gene expression patterns. On the other hand, by treating each niche as the basic unit, ONTraC generates more robust results.

Furthermore, we benchmarked ONTraC against two recently developed methods, SpaceFlow [7] and spatialPCA [19], that extend the pseudotime framework by incorporating spatial cell-neighborhood information for cell-state embedding. To fully preserve the spatial cell-neighborhood information, we applied these methods only to the whole dataset (Fig. 1e and Additional file 1: Fig. S2). Both methods performed better than the pseudotime methods above, with Spearman correlation at 0.77 and 0.78, respectively (Fig. 1e), suggesting that incorporating spatial information is beneficial for spatial trajectory analysis. However, they still cannot correctly map lineage 2 cells onto the spatial trajectory (Additional file 1: Fig. S2).

To test the performance of ONTraC on more complex scenarios, we generated three additional simulated datasets to explore various spatial patterns and trajectory topologies (linear, nonlinear, and segregated configurations) and cell lineage relationship (multiple bifurcations and disconnected cell lineages) (Methods; Additional file 1: Fig. S4, Additional file 2: Table S1, and Additional file 3: Table S2). For each simulated dataset, we benchmarked the performance of ONTraC against existing methods and found ONTraC outperformed existing methods for all datasets (Additional file 1: Fig. S4e). Taken together, these analyses indicate that ONTraC is more accurate than existing methods in spatial trajectory reconstruction.

### Dissecting the spatial structure of the mouse motor cortex using niche trajectory analysis

We applied ONTraC to analyze a MERFISH dataset from the mouse motor cortex, which includes 64 tissue slices and contains approximately 280,000 cells characterized by a 258-gene panel [24]. The study identified 23 transcriptional subclasses, several of which exhibit distinct spatial patterns (Fig. 1f). Interneurons (IT) are distributed across all cortical layers, with each associated with a distinct IT subclass. The vascular leptomeningeal cells (VLMCs) demarcate a thin layer at the border of the cortex. We treated each subclass as a “cell type,” while aggregating the remaining cells under the cell type “other.” Using information from all 24 cell types (i.e., 23 subclasses + other), we constructed the niche trajectory by applying the ONTraC workflow. The resulting NT faithfully reveals the underlying layer structure (Fig. 1g), starting from the outer layer and progressively moving inward. The sharp transition between the layer boundaries is also preserved. The cell-type composition varies continuously along the NT, with each layer-specific cell type enriched within a narrow section (Fig. 1h). On the other hand, non-neuronal cell types such as astrocytes and microglia spread broadly (Additional file 1: Fig. S5a).

As an additional validation, we tested whether the cell-level NT scores correlate with cortical depths. A similar analysis was performed in the original paper and resulted in high correlation [24], but the analysis was limited to IT neurons. In contrast, our ONTraC included all cells regardless of their cell types (see Methods for details). We observed a high degree of correlation between the cell-level NT scores and cortical depths (Additional file 1: Fig. S5b). For over 87% (56/64) of the tissue sections, the Spearman correlation between cell-level NT scores and cortical depths is 0.90 or higher. However, a small number of samples exhibited significantly lower correlations. For example, in the Mouse2 Slice99 sample, the correlation was only 0.76. To understand the underlying cause, we compared the cell-type distribution between the Mouse1 Slice301 (with highest correlation = 0.99), Mouse2 Slice169 (with medium correlation = 0.97), and Mouse1 Slice99 (with lowest correlation = 0.76). We noticed that the VLMC layer is completely included in Mouse1 Slice301 and Mouse2 Slice169 but is partially missing in Mouse2 Slice99 (Fig. 1f). The discrepancies between cell-level NT scores and cortical depths are caused by the error in the cortical depth calculation method, which is sensitive to the missing VLMC layer information (Additional file 1: Fig. S5b, insets). On the other hand, our ONTraC method is reference free, thereby providing a more robust approach for evaluating cortical depth. To test the robustness of ONTraC against parameter settings in real data, we repeated ONTraC analysis using various parameter settings and found the results are generally in good agreement (Additional file 1: Fig. S3).

To benchmark the performance of ONTraC with existing methods, we used the cortical depth as a proxy to the ground-truth. We reasoned that, despite its limitations, the cortical depth serves as an independent reference, and that any ascertainment error should equally impact the performance evaluation of all methods. We compared the performance of ONTraC with existing methods. In all cases, ONTraC outperforms other methods (Additional file 1: Fig. S6).

Of note, in the original study [24], the authors also applied pseudotime analysis and showed high correlation between pseudotime and cortical depths. However, their findings do not contradict with our results, because their analysis was done by using IT neurons, which are known to have defined layer specific distributions. On the other hand, in the above benchmark analysis, we applied pseudotime analysis to the whole dataset. As shown in our analysis of simulated dataset 1, the performance of pseudotime analysis strongly depends on the choice of cell lineages in the analysis (Fig. 1e). To confirm this interpretation was correct, we compared the cell-level NT scores obtained from ONTraC with the pseudotime values for IT neurons and found that the results from these two approaches were indeed highly correlated (*R* = 0.92) (Additional file 1: Fig. S5c). By including all the cells in spatial trajectory analysis, ONTraC provides an unbiased view of the tissue microenvironment.

### ONTraC analysis identifies tissue microenvironment associated cell-state variations in developing dorsal midbrain

Tissue microenvironment plays a distinctly important role during development, where cells that are initially identical undergo divergent paths during differentiation. To investigate the role of the tissue microenvironment in mediating cell state changes, we analyzed a public stereo-seq dataset from mouse embryo development [25]. This dataset includes whole-embryo, transcriptome-wide, single-cell gene expression profiles across eight time points between E9.5 and E16.5. We focused on a subset that was analyzed extensively in the original study, containing 27k segmented cells from the dorsal midbrain region across three time points: E12.5, E14.5, and E16.5. We annotated cell types following the same procedure as in the original study [25] (see Methods). Consistent with the original study, we identified ten cell types, including radial glial cell (RGC), glioblast (GioB), neuroblast (NeuB), glutamatergic neuroblast (GluNeuB), glutamatergic neuron (GluNeu), GABAergic neuron (GABA), basal, fibroblast (Fibro), endothelial (Endo), and erythroid (Ery) cells (Additional file 1: Fig. S7). While the original study also detected a small cell cluster annotated as microglia, our analysis did not identify such a cluster. This discrepancy is likely due to the intrinsic uncertainty associated with rare cell type detection.

**Fig. 2:**
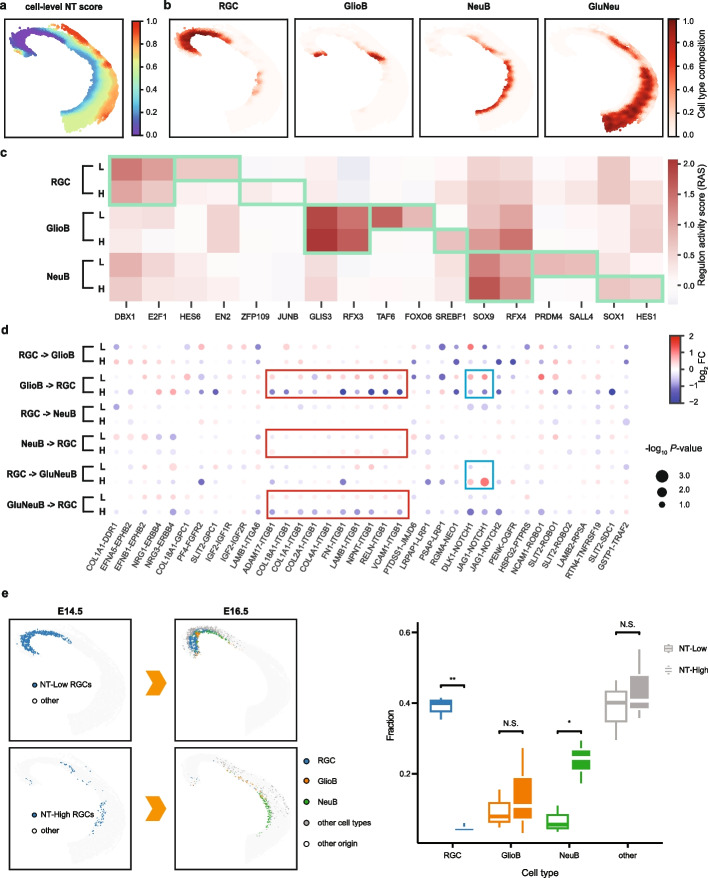
ONTraC analysis reveals tissue-microenvironment-mediated cell-state changes in E14.5 mouse dorsal midbrain. **a** Spatial distribution of cell-level NT scores. **b** Spatial distribution of representative cell types. **c** Changes in regulon activity associated with cell type and microenvironment. Green boxes highlight cell-type or microenvironment-specific high regulon activities (H: NT-High; L: NT-Low). **d** Cell-type-pair dependent ligand-receptor activities in NT-High (H) and NT-Low (L) microenvironments. ITGB1 and NOTCH1 associated signals are highlighted by red and blue boxes, respectively. **e** Moscot analysis mapping E14.5 NT-Low and NT-High RGCs to putative offspring cells in E16.5. Left: spatial locations of original and mapped cells; right: box plots showing the cell-type composition of putative offspring cells of E14.5 NT-Low and NT-High RGCs. Statistics are based on all three E16.5 mouse samples. N.S. (non-significant); * (*P* value < 0.05); ** (*P* value < 0.01)

To identify changes in tissue structure across developmental stages, we trained a common ONTraC model for all the samples to ensure the resulting NT scores are directly comparable. Within each sample, the niche trajectory progresses continuously from caudal to rostral and from ventral to dorsal areas (Fig. 2a, Additional file 1: Fig. S8). Along the niche trajectory, the most enriched cell type transitions from RGCs to increasingly differentiated cell types (Fig. 2b, Additional file 1: Fig. S9). Comparing the NT score distributions across different samples, we noted a significant increase as development progresses (Additional file 1: Fig. S10). Altogether, these results suggest that the niche trajectory delineates a path from undifferentiated to increasingly more differentiated tissue microenvironment.

Among these cell types, RGCs are especially notable due to their ability to migrate and differentiate into either neuronal or glial cells during embryonic development. The spatial distribution of RGCs exhibits distinct spatiotemporal patterns (Fig. 2b and Additional file 1: Fig. S9). At E12.5, RGCs occupy nearly the entire dorsal midbrain but become increasingly spatially restricted during development. At E14.5, they are distributed along a narrow streak at the ventral edge. By E16.5, the RGCs are confined to the caudal domains.

To investigate whether changes in the tissue microenvironment play a role in mediating the gene expression programs in RGCs, we focused on the E14.5 time point, when the RGCs are distributed across a wide range along the niche trajectory (Fig. 2a, b), indicating exposure to diverse tissue microenvironments. By using a metacell-based strategy (see Methods), we identified 109 genes whose expression levels are significantly correlated with cell-level NT scores (*P* value < 0.01 and absolute value of Pearson correlation coefficient > 0.4), including 95 positively and 14 negatively associated genes (Additional file 4: Table S3). Among these genes, several have been previously implicated in either stem cell renewal or neurogenesis, such as *Ppia* [26], *Ccnd2* [27], *Efna5* [28], *Cdkn1c* [29], *Phf21b* [30], and *Dcc* [31] (Additional file 1: Fig. S11a). This observation is further supported by gene set enrichment analysis (GSEA) [32]; we identified found several enriched Gene Ontology (GO) terms that are related to stem cell renewal or neurogenesis, such as “DNA replication initiation” (NES = − 2.36, *P* value < 1 × 10^−308^), “Non-canonical WNT signaling pathway via MAPK cascade” (NES = 1.80, *P* value = 2.0 × 10^−3^), and “Planar cell polarity pathway involved in neural tube closure” (NES = 1.77, *P* value = 1.0 × 10^−2^) (Additional file 1: Fig. S11b).

To gain mechanistic insights, we performed gene regulatory network (GRN) analysis using the SCENIC workflow [33]. To identify niche trajectory associated GRN changes, we divided the E14.5 RGCs into two subgroups based on their cell-level NT scores: NT-Low (< 0.17) and NT-High (≥ 0.17). Consistent with the original study [25], our analysis identified several known cell-type-specific regulators, such as DBX1 and E2F1 for RGCs, GLIS3 and RFX3 for glioblasts, and SOX9 and RFX4 for neuroblasts (Fig. 2c and Additional file 1: Fig. S12). Whereas the original study only examined GRN differences between cell types, here we were able to further investigate within cell-type variations by leveraging the NT structure. By comparing the RAS scores associated with NT-Low and NT-High subgroups, we identified several regulons whose activities change significantly along the NT, such as HES6 and EN2 (in RGCs), FOXO6 and SERBF1 (in GlioB), and PRDM4 and SOX1 (in NeuB) (Fig. 2c). This finding suggests that these regulators may play a role in fine-tuning the timing of cell differentiation in a niche-dependent manner.

Next, we performed cell–cell interaction analysis using the Giotto pipeline [34, 35] and compared the patterns between NT-Low and NT-High niches (see Methods). Cell proximity analysis indicates that RGCs tend to be isolated from other cell types in NT-Low niches, whereas in NT-High niches, they are more likely to interact with glioblasts, neuroblasts, and glutamatergic neuroblasts (Additional file 1: Fig. S13). Furthermore, ligand-receptor pair analysis identified significant changes between NT-Low and NT-High niches (Fig. 2d) associated with NOTCH1 and integrin beta 1 (ITGB1) signaling pathways, both are known to regulate radial glia differentiation [36, 37].

To further investigate the connection between NT and cell fate changes, we linked cells obtained from different time points using the Moscot procedure [38] (see Methods). We compared the predicted offspring associated with NT-Low and NT-High RGCs and observed significant differences (Fig. 2e). Whereas the NT-Low RGC offspring cells tend to retain the RGC identity, the NT-High RGC offspring cells are more differentiated (Fig. 2e). Taken together, the above analyses strongly suggest that ONTraC is a useful tool for dissecting the role of tissue microenvironment changes in mediating transcriptional changes and cell-fate decisions.

### ONTraC identifies cell type trajectories in tumor microenvironment

Tumor microenvironment often contains a higher degree of cellular heterogeneity and more complex spatial structure. To test the utility of ONTraC for analyzing tumor microenvironment, we analyzed a publicly available human breast cancer Xenium dataset [39] (Methods). This dataset contains diverse non-tumor cell types, such as endothelial, stromal, and immune cells, as well as ductal cancer and invasive tumor cells (Additional file 1: Fig. S14). The niche trajectory delineates a path that reflects the spatial transition from the tumor core to surrounding tissues. Of note, the tumor core is enriched with invasive tumor cells, whereas the tumor boundary is enriched with proliferative tumor cells (Additional file 1: Fig. S14), suggesting the tumor microenvironment may play an important role in modulating cancer cell behavior. In addition, our analysis showed that the immediate tumor surrounding tissue is enriched in immune cells, whereas the more distal regions are enriched in endothelial and stromal cells. Similar spatial organization patterns have also been found in other cancer types [40].

### Comparison with spatial domain analysis

To illustrate the difference between ONTraC and traditional spatial domain analysis methods, we re-analyzed these three real datasets using GraphPCA [11] as a representative method (Methods) (Additional file 1: Fig. S15a). For the MERFISH data, GraphPCA can also accurately recapitulate the cortical layer structure (Additional file 1: Fig. S15b); however, it cannot capture intra-layer variations revealed by ONTraC analysis. As noted by the authors of the original paper, even some commonly used layer-specific markers show intra-layer variations, such as *Rspo1* (a L4 marker) and *Fezf2* (a L5 marker) (Additional file 1: Fig. S16a). Such intra-layer variations are accurately detected by ONTraC (Additional file 1: Fig. S16b), while GraphPCA cannot detect such variations due to the limitation of spatial domain analysis (Additional file 1: Fig. S16c).

Similarly, for the stereo-seq dataset, while both GraphPCA and ONTraC can detect the overall TME variations, ONTraC reveals more refined spatially associated variations as compared to GraphPCA (Fig. 2 and Additional file 1: Fig. S15). To gain further insights, we examined the RGC gene expression patterns within domain 3 identified by GraphPCA, which straddles across a relatively wide range along the niche trajectory. In the above, we showed that several genes that are significantly associated with spatial trajectory yield by ONTraC (Additional file 1: Fig. S11a). Here, we compared the gene expression levels between NT-High and NT-Low RGCs in domain 3, using *Efna5* and *Dcc* as representative examples. Both genes were differentially expressed between NT-High and NT-Low RGCs even within the same spatial domain (*Efna5*, *P* value = 2.6 × 10^−4^; Dcc, *P* value = 1.9 × 10^−2^; two-sided *t*-test), suggesting that ONTraC is more sensitive than spatial domain analysis in detecting gene expression variations. Finally, we compared the results of ONTraC and GraphPCA on the Xenium breast cancer dataset. As described above, ONTraC captures the smooth spatial transition from the invasive to proliferative tumor cells, followed by immune cells and stromal area (Additional file 1: Fig. S14). In contrast, GraphPCA groups all the cancer cells in one domain (Additional file 1: Fig. S15c), reducing the identification of subtle cell type trajectories across the sample. Taken together, these analyses strongly indicate ONTraC has advantages over traditional spatial domain analysis in detecting spatially continuous variations of the tissue microenvironment.

## Discussion

We have developed ONTraC as a new framework for constructing spatial trajectories. This framework is generally applicable. While the above analysis is focused on spatial transcriptomics data, the ONTraC framework is generally applicable because it only requires cell-type and spatial information as the input. A key feature of ONTraC is that it treats a niche, rather than a cell, as the basic unit. While the use of niche structure in spatial analysis is not new [1, 9], ONTraC differs from previous approaches in that it explicitly models the hierarchy of niche-level and cell-level properties. Specifically, the niche-level properties are shared among all cells within a niche, rather than being limited to any specific cell type or cell state. Of note, while some cells may change identity along the niche trajectory, such changes are neither required nor implicated in the niche trajectory analysis. On the other hand, cell-level properties are maintained and can be compared at the single-cell resolution. This strategy naturally incorporates local cellular heterogeneity as a source of spatial variation at the niche-level, while simultaneously providing a common framework to study coordinated responses from multiple cell types within the tissue microenvironment. Through extensive benchmark analyses using both simulated and real datasets, we have shown that ONTraC outperforms existing methods for spatial trajectory analysis. Moreover, the spatial distribution of NT scores serves as a useful guide to systematically study the impact of the tissue-microenvironment changes on mediating cell-state dynamics and cell–cell communications. Compared to the discrete spatial domain analysis, the continuous NT scores are more sensitive for detecting refined spatial variations, as evidenced by our comparative analysis with GraphPCA.

Inspired by previous work from several groups [5, 7, 9], ONTraC utilizes a GCN modeling framework to integrate spatial and cell-type composition information. In previous work, the use of GCN was limited to characterizing spatial domains, but here we have extended its use for constructing spatial trajectories. Spatial information is preserved throughout the ONTraC workflow, as all the analyses are supported by the underlying spatial network structure. The loss function is designed to properly balance the conflicting demands for preserving both node property similarity and spatial continuity. Our analyses show that ONTraC performs well in analyzing real biological datasets in various contexts.

ONTraC has several limitations. First, since it requires cell-type annotation information as input, ONTraC is designed primarily for analyzing spatial data with single-cell resolution. Second, the granularity of cell type annotations may have a significant impact on the prediction outcome. Third, the output of ONTraC is an unbranched trajectory, which may be over-simplified for representing the spatial structure of a complex tissue. Lastly, the current ONTraC workflow is not yet scalable for analyzing large datasets (Additional file 5: Table S4). Future extensions are needed to overcome these limitations.

Finally, there is a growing interest in integrating spatial omic and pathological information to study human diseases [41]. Such integrations could lead to comprehensive understandings of the disease progression process and further provide mechanistic insights. Niche trajectory analysis may provide a useful tool for such integrations.

## Conclusions

Taken together, our work shows that niche trajectory analysis provides an innovative and useful framework for analyzing the structure and function of the tissue microenvironment and that ONTraC is an effective method for constructing niche trajectories.

## Methods

### The ONTraC workflow

The ONTraC workflow includes the following steps: 1. Constructing a niche network; 2. Using GCN to encode input data in a low-dimensional feature space; 3. Using graph pooling to identify niche clusters and their spatial relationship; 4. Finalizing niche trajectory construction and NT-score evaluation (Fig. 1a and Additional file 1: Fig. S1). The details are described in the following.

#### Step 1: constructing a niche network

A niche is defined as a multicellular, spatially localized region where different cell types may coexist and interact. Although the general workflow is sufficiently flexible to accommodate variations in the precise definition of niches, the analyses in this paper were conducted using the following procedure to ensure reproducibility: Each niche is anchored at a single cell, which is referred to as the anchoring cell, and including its *k*-nearest neighbors (kNN) (default $k=50$) according to physical distance. A niche network is created by connecting pairs of niches whose anchoring cells are mutual nearest neighbors. The edge relationship in the niche network is represented by the binary adjacency matrix $A=\left({a}_{ij}\right).$

Since neighboring niches may overlap, each cell is typically included in multiple niches. The degree of association between a cell *i* and a niche* j* that contains it is quantified by the cell-niche association score, which is defined by ${w}_{ij}={e}^{-\left({d}_{ij}^{2}/{\sigma }_{j}^{2}\right)}$, where ${d}_{ij}$ denotes the physical distance between the cell *i* and the anchoring cell of niche *j*, and ${\sigma }_{j}$ represents a niche-specific normalizing factor. By default, the value of ${\sigma }_{j}$ is set to be the spatial distance between the anchoring cell and its 20 th nearest neighbor. Based on its cell-type composition, a niche *j* is assigned with a numerical vector ${\text{v}}_{{\varvec{j}}}=\left({v}_{jm}\right)$, whose values are given by ${v}_{jm}=\sum_{i}{w}_{ij}{I}_{im}/\sum_{i}{w}_{ij}$, where ${I}_{im}$ is a binary indicator of whether the cell *i* belongs to the cell-type *m*.

#### Step 2: using GCN to encode input data in a low-dimensional feature space

We use a two-layer GCN model to integrate spatial and cell-type composition information, which can be mathematically represented as:

$${X}_{t}={G}_{t}({X}_{t-1}, \tilde{A} )=SeLU((\tilde{A} +I){X}_{t-1}{W}_{t})$$

where ${X}_{t}$ (*t* = 1 or 2) represents the low-dimensional embedding vectors (dimension = 4 by default) at the *t*th layer, while ${X}_{0}$ represents the input cell-type composition vectors ${\text{v}}_{{\varvec{j}}}$ obtained from step 1. $\tilde{A} ={D}^{-\frac{1}{2}}A{D}^\frac{1}{2}$ represents the normalized adjacency matrix, with $D=diag(A)$, and ${W}_{t}$ represents the trainable parameters. The model training process is described later.

#### Step 3: using graph pooling to identify niche clusters and their spatial relationship

In this step, we use a modified graph pooling approach [21] to convert the original spatial network into a network of niche clusters. To this end, we probabilistically cluster niches based on the embedding vector values generated in step 2. The main output is a matrix $C=\left({c}_{jk}\right)$, whose values ${c}_{jk}$ represent the probabilistic assignment of a niche *j* into cluster *k*. For this purpose, we use a linear neural network model, $C=$ softmax ${(\beta X}_{2}{W}_{C}),$ where $\beta$ is a tuning parameter set to a default value of 0.03, and ${W}_{C}$ contains trainable parameters.

Next, we perform graph pooling by collapsing the niche network to a niche cluster network. For each pair of niche clusters $k$ and $l$, the corresponding edge connectivity is given by $E(k,l)=\sum_{i}\sum_{j}{c}_{ik}{a}_{ij}{c}_{jl}$, where $\left({a}_{ij}\right)$ and $\left({c}_{jk}\right)$ represent the adjacency matrix and the niche cluster assignment matrix, respectively. The edge connectivity is further normalized by $\widetilde{E}(k,l)=E(k,l)/\left(\sum_{i}E(k,i)*\sum_{i}E(i,l)\right)$.

#### Step 4: finalizing niche trajectory construction and NT-score evaluation

In the following, we use *N*, *M*, and *K* to represent the total number of niches, cell types, and niche clusters, respectively. To construct the niche trajectory, we search for the optimal ordering of niche clusters: ${p}_{1}\to {p}_{2}\to \dots \to {p}_{K}$ that maximizes the total edge connectivity, which is defined by $\sum_{{p}_{k}=1}^{K-1}\widetilde{E}\left({p}_{k},{p}_{k+1}\right)$. Then, the niche cluster ${p}_{k}$ is assigned a score $s\left({p}_{k}\right)=\left(k-1\right)/\left(K-1\right)$. Next, we map each niche* j* along the trajectory by considering its probabilistic assignment to each niche cluster. The corresponding coordinate, called the NT score, is defined as:

$${s}_{j}=\sum_{k=1}^{K}{c}_{jk}s(k)$$

The range of NT scores is between 0 and 1. Finally, to account for the varying degrees of association between cells and niches, we further define the cell-level NT score for each cell* i* as follows:

$${\widetilde{s}}_{i}=\frac{{\sum }_{j}{w}_{ij}{s}_{j}}{{\sum }_{j}{w}_{ij}}$$

The cell-level NT score is used to stratify cells to study within cell-type variations mediated by tissue microenvironment changes.

#### Model training

The ONTraC workflow contains two sets of trainable parameters ${W}_{t}$ (step 2) and ${W}_{C}$ (step 3). These parameters are trained to optimize spatial and cell-type composition continuity. Toward this end, we design a loss function that contains three terms representing modularity, purity, and regularization.

The modularity loss term is given by:

$${L}_{m}=-Q=-Tr\left({C}^{T}BC\right)/2N$$

where $Q$ is known as the modularity [42], and *C* is the probabilistic cluster assignment matrix defined in step 3. *B* is the modularity matrix, which is defined by $B=A-\frac{d{d}^{T}}{2N}$, where *d* is the node degree vector. This term is included to enhance modularity.

The purity loss term is given by:

$${L}_{p}=\frac{1}{N\times M}\sum_{j}\sum_{m}{\left({v}_{jm}-\sum_{k}{c}_{jk}{v}_{km}^{*}\right)}^{2}$$

where ${\text{v}}_{{\varvec{j}}}=\left({v}_{jm}\right)$ represents the cell-type composition vector associated with niche *j*, and ${\text{v}}_{k}^{*}=\left({v}_{km}^{*}\right)$ represents the average cell-type composition vector within niche cluster *k*. This term is introduced to enhance feature similarity within each niche cluster.

Finally, the regularization loss term is given by:

$${L}_{r}=\frac{\sqrt{K}}{N(\sqrt{K}-1)}\sqrt{\sum_{j}{\left(\sum_{k}{c}_{jk}\right)}^{2}}$$

This term is introduced to regularize the distribution of cluster sizes [21].

Taken together, the overall loss function is given by

$${L}_{total}={\lambda }_{m}\cdot {L}_{m}+{{\lambda }_{p}\cdot {L}_{p}+\lambda }_{r}\cdot {L}_{r}$$

For the analyses in this paper, we use the following parameter setting: ${\lambda }_{m}=0.3$, ${\lambda }_{p}=300,$ and ${\lambda }_{r}=0.1.$ The values of the trainable parameters ${W}_{t}$ and ${W}_{C}$ are obtained using Adam optimizer [43] with 0.03 as the learning rate. The model is implemented using PyTorch (https://pytorch.org/) and PyTorch Geometric (https://github.com/pyg-team/pytorch_geometric) and carried out on a NVIDIA H100-80GB NVlinked GPU. In our analysis, we set the batch size to 5 (stereo-seq dataset) and 10 (MERFISH dataset) with a maximum of 1000 epochs.

### Simulated spatial transcriptomic data generation

Simulated spatial transcriptomic data were generated by using two steps: 1. single-cell gene expression data matrix generation and 2. spatial coordinate assignment. As a proof-of-concept, we began by generating an initial simulated dataset (simulated dataset 1) with idealized cell lineage relationship and spatial pattern. Single-cell gene expression data were generated using *dyngen* [22] with the default bifurcating backbone, which contains two cell lineages diverging from a single bifurcation point. A total of 1000 cells were sampled in 10 batches. Based on their relative positions along the trajectory, the cells were assigned to 10 different cell-types (A–J), with cell types A–G mapped to lineage 1 and cell types H–J to lineage 2 (Additional file 1: Fig. S4). The simulated gene expression profiles contain 135 genes, including 35 transcription factors, 50 target genes, and 50 housekeeping genes (Additional file 2: Table S1; Additional file 3: Table S2).

Second, each cell is assigned to a spatial location as follows: Each cell *i* in lineage 1 is mapped to a location $({x}_{i}, {y}_{i})$, which is randomly chosen from the circle ${x}^{2}+{y}^{2}={r}_{i}^{2}$. The radius ${r}_{i}$ is proportional to the simulated time ${t}_{i}$ obtained from *dyngen* output and rescaled so that its maximum value is 1. Each cell in lineage 2 is mapped to a random location in the area defined by ${x}^{2}+{y}^{2}\le 1$. As the niche-level, the spatial trajectory progresses radially from the center of the circle, and the ground-truth cell-level NT score is equal to ${r}_{i}$ for all cells. While the ordering of lineage 1 cells along the spatial trajectory maintains their lineage relationship, the spatial distribution of lineage 2 cells is unrelated to their lineage relationship.

Three additional spatial transcriptomic datasets were simulated using a similar strategy but tailored to more complex spatial patterns and trajectory topologies. Among these, simulated datasets 2 and 3 were based on a common single-cell gene expression dataset but have different spatial patterns (Additional file 1: Fig. S4; Additional file 2: Table S1; Additional file 3: Table S2). The single-cell gene expression dataset was simulated using the binary tree backbone in *dyngen*. A total of 500 cells were simulated in 10 batches. The cells were sampled from three different lineages: lineage 1 spans the entire length of the simulation time and consists of cell types A to F, lineage 2 branches off from lineage 1 from an earlier simulation time and consists of cell types G to H, and lineage 3 branches off at a later simulation time and consists of cell types I and J. To simulate spatial transcriptomics data, the cells were mapped to spatial locations using two different spatial patterns: linear (simulated dataset 2) vs nonlinear (simulated dataset 3). The linear pattern was generated by distributing lineage 1 cells along the x-axis in accordance with their lineage relationship, whereas the nonlinear pattern was generated by assigning spatial coordinates based on a quadratic function (Additional file 1: Fig. S4; Additional file 2: Table S1; Additional file 3: Table S2). On the other hand, the spatial coordinates of lineage 2 and 3 cells were randomly assigned.

Simulated dataset 4 was generated by using a different trajectory topology. Again, the first step was to simulate a single-cell gene expression dataset using *dyngen*, but here we used the disconnected backbone to simulate two unrelated cell lineages. Lineage 1 cells exhibited earlier simulation time overall than lineage 2 cells. This dataset contains 454 cells in total. Cells from the two lineages were mapped mutually exclusive regions, with lineage 1 cells being mapped toward the middle of the simulated tissue whereas lineage 2 cells mapped toward the periphery (Additional file 1: Fig. S4; Additional file 2: Table S1; Additional file 3: Table S2). The assigned x-coordinates were proportional to the absolute value of the simulation time, and y-coordinates were random.

### Benchmark analysis

To benchmark the performance of ONTraC, we considered five existing methods for comparison and applied each method to the simulated dataset, including three pseudotime analysis methods: destiny (v3.16.0) [14], Monocle 3 (v1.3.7) [17], and TSCAN (v2.0.0) [15], as well as two methods that further incorporate spatial information: SpaceFlow (v1.0.4) [7] and SpatialPCA (v1.3.0) [19]. For each pseudotime analysis method, three sets of results were generated, corresponding to all cells, lineage 1 cells, and lineage 2 cells, respectively. On the other hand, SpaceFlow and SpatialPCA were applied only to analyze all cells to faithfully represent the spatial information.

For destiny, dimensionality reduction was performed using “*DiffusionMap*” with default parameters, and pseudotime values were generated using the “*DPT*” function. For Monocle 3, dimensionality reduction was achieved using the “*reduce_dimension*” function with the UMAP method, and pseudotime values were obtained by using the “*pseudotime*” function. The root cell was selected to be the true starting cell along the trajectory. For TSCAN, pseudotime values were obtained by sequentially applying the “*preprocess*,” “*exprmclust*,” and “*TSCANorder*” functions.

For SpaceFlow, pseudotime values were obtained by sequentially applying the “*preprocessing_data*,” “*train*,” “*segmentation*,” and “*pseudo_Spatiotemporal_Map*” methods with default parameters. For SpatialPCA, spatial principal component analysis was performed by sequentially applying the “*SpatialPCA_buildKernel*,” “*SpatialPCA_EstimateLoading*,” and “*SpatialPCA_SpatialPCs*” functions. Cell clusters were obtained using the “*walktrap_clustering*” function with parameters “clusternum = 6, knearest = 32.” The pseudotime was obtained using Slingshot (v2.10.0) following the tutorial of SpatialPCA.

To benchmark its running time and computer memory, we tested ONTraC and all other methods under the same environment (Linux RHEL 9.4, up to 12 Intel Xeon Platinum 8568Y + Processor cores, 9.7 GB memory, and 1 H100-80GB NVlinked GPU) using 4 simulated dataset (1 sample, 500–1000 cells), stereo-seq mouse midbrain dataset (5 samples, 27k cells), and MERFISH mouse cortex dataset (64 samples, 280k cells). For the latter, we used a batch training method (batch size = 10) to reduce computation and memory burden. The actual usage of CPU, memory, GPU, GPU memory, and running time are presented in Additional file 5: Table S4. To be noted, ONTraC can analyze MERIFSH data with 280k cells on NVIDIA V100, GeForce RTX 3090/4080 or higher models of GPU since it only requires 14 GB GPU memory. In future work, we will continue working to enhance scalability.

### Analysis of mouse cortex MERFISH data

Processed mouse motor cortex MERFISH data, including count matrix, cell type annotation, spatial location, and other meta information, were obtained from Brain Image Library [44]. For cell type annotation, we treated each of the 23 transcriptome subclasses identified by the original study as a distinct cell type. Additionally, another cell type, termed “other,” was used to annotate those cells that do not fall into any of these transcriptional classes. The cortical depth was calculated by using the VLMC cell layer as the reference. However, a small number of annotated VLMC cells were located in the interior of the cortex layers, which likely resulted from annotation error. After removing these misannotated VLMC cells, we calculated the cortical depth for each cell as its distance to the nearest VLMC cell. We performed pseudotime analysis by using the destiny (v3.16.0) using the same setting as in the benchmark analysis. Only IT neurons were included in this analysis as in the original study.

### Analysis of mouse embryo dorsal midbrain stereo-seq data

Mouse embryo stereo-seq data were downloaded from the MOSTA website (https://db.cngb.org/stomics/mosta/download/). We analyzed the dorsal midbrain subset corresponding to the following five samples: E12.5 E1S3, E14.5 E1S3, E16.5 E1S3, E16.5 E2S6, and E16.5 E2S7. Segmented cell locations and the single-cell resolution gene expression profiles were generated by the authors. Because the authors did not provide cell-type annotation information, we regenerated the cell-type labels by following the same procedure as described by the authors. Briefly, we normalized gene expression using SCTransform [45] (as implemented in Seurat v4.3.0) and removed the batch effect using harmony (v1.2.0) [46]. This was followed by dimension reduction using UMAP and Leiden clustering [47]. The identified clusters were annotated using known cell-type specific marker genes. Comparing the UMAP (Additional file 1: Fig. S7) with the original study (Fig. 5 A in ref. [25]) shows similar structures, indicating the overall cell-type structure is reproduced. The remaining discrepancy is likely due to the difference between our parameter setting and software version and the original study.

To identify genes whose expression level changes significantly along the niche trajectory, we used a metacell-based correlation analysis described as follows. The E14.5 RGCs were ordered based on their corresponding cell-level NT score values, and then divided into subgroups each containing 10 cells. For each subgroup, the cell-level gene expression profiles were aggregated, thereby creating a metacell. Then, we evaluated the Pearson correlation coefficients between metacell gene expression and NT scores. Significantly associated genes were defined as *P* value < 0.01 and absolute value of correlation coefficient > 0.4. Gene set enrichment analysis was carried out by using the GSEApy (v1.1.5) [48].

To gain mechanistic insights, we further performed gene regulatory network, cell–cell interaction, and spatiotemporal mapping analyses as described below. For these analyses, we divided E14.5 RGC cells into two subgroups by thresholding the cell-level NT scores (cutoff = 0.17). These subgroups are named NT-Low or NT-High, respectively. Using a different cutoff value produced similar results (Additional file 1: Fig. S17).

#### Gene regulatory network analysis

Transcription factor regulons were identified using the SCENIC workflow [33] as implemented in pySCENIC v0.12.1. A gene co-expression network was inferred using the “grn” module with default parameters. Motif enrichment analysis for each gene co-expression module was predicted using the “*ctx*” module combined with the pre-computed SCENIC + database (mm10 refseq_r80 v10). Regulon activity scores (RAS) were quantified at the single-cell level using AUCell and then normalized by transforming them into *z*-scores within each developmental stage. Regulon specificity scores (RSS) were calculated using the “*regulon_specificity_scores*” function based on the algorithm described in ref. [49].

#### Cell–cell interaction analysis

The Giotto Suite package [34, 35] is used for cell proximity and cell–cell interaction analyses. Spatial networks were constructed using the “*createSpatialNetwork*” function with the parameter setting “*method* = *‘knn’, k* = *5*.” Cell proximity analysis was performed using the “*cellProximityEnrichment*” function with parameter “*number_of_simulations* = *1000*.” Ligand-receptor enrichment analysis was performed by using the “*exprCellCellcom*” and “*spatCellCellcom*” functions with the parameter setting “*random_iter* = *1000, adjust_method* = *‘fdr’*.”

#### Spatiotemporal mapping of cells across different samples

The Moscot pipeline [38] was used for spatiotemporal mapping between E14.5 and E16.5 dorsal midbrain stereo-seq samples with default parameter setting. The main output of Moscot is a learned coupling matrix that probabilistically connects cells from an early to a late time point. For each RGC from the E14.5 sample, we identified its putative offspring cells in the E16.5 sample as the set of five cells with the highest non-zero coupling probabilities. Depending on their cell origin, a putative offspring cell is labeled either as “NT-Low offspring” or “NT-High offspring.” To eliminate uncertainty, the cells identified as the putative offspring of both NT-Low and NT-High RGCs are excluded from further study.

### Analysis of the human breast cancer Xenium dataset

We downloaded the replicate 1 from sample 1 of the human breast cancer Xenium dataset [39] from 10X Genomics website (https://www.10xgenomics.com/products/xenium-in-situ/preview-dataset-human-breast). We used the Giotto Suite package v4.1.4 [35] to remove the cells with less than 100 genes and to select the region between the x coordinates 3000–4500 and the y coordinates 1000–2500. The final selection contains 6912 cells. We used the cell type annotation provided by the authors and the spatial coordinates to run the ONTraC analysis on the selected region.

### Spatial domain analysis

We utilized GraphPCA (v0.1.3) [11] to generate spatial domain results, using gene counts and spatial coordinates as input. Parameters were set to default, except for the number of domains: 6 for most datasets and 4 for the Xenium breast cancer dataset. For the MERFISH mouse cortex dataset, we analyzed Mouse1 Slice301, Mouse2 Slice169, and Mouse2 Slice99, excluding other slices to prevent memory overflow in the GraphPCA pipeline. Reproducible code is available for further details.

## Supplementary Information

Additional file 1: Supplementary figures.

Additional file 2: Table S1 Gene expression matrix for the four simulated datasets.

Additional file 3: Table S2 Metadata and spatial coordinates of the cells for the four simulated datasets.

Additional file 4: Table S3 Genes whose expression levels in E14.5 mouse dorsal midbrain RGCs are significantly correlated with cell-level NT scores.

Additional file 5: Table S4 Computer running time and memory usage for ONTraC analysis of different datasets.

## Data Availability

ONTraC The ONTraC method is implemented as a Python package, publicly available at GitHub (https://github.com/gyuanlab/ONTraC) and Zenodo [50]. The source code is released under the MIT License. A website is available at https://ontrac-website.readthedocs.io/ with detailed instructions for installation, execution, and data analysis tutorials. Datasets Simulated Datasets Generation of the simulated spatial transcriptomic data is described in Methods, and the simulated datasets are available for download from the Zenodo repository [51]. Public Datasets Mouse embryo Stereo-seq data were obtained from the MOSTA website (https://db.cngb.org/stomics/mosta/download/). Processed mouse motor cortex MERFISH data, including count matrix, cell type annotation, spatial location, and other meta information, were obtained from Brain Image Library [44]. Human breast cancer Xenium dataset [39] from 10X Genomics website (https://www.10xgenomics.com/products/xenium-in-situ/preview-dataset-human-breast) Reproducible codes Codes for reproducing all results presented in this paper are available at GitHub (https://github.com/gyuanlab/ONTraC_paper). This information is included in the Zenodo [50].
