# Supplementary material for: ONTraC characterizes spatially continuous variations of tissue microenvironment through niche trajectory analysis
Source: Genome Biol. 2025 May 8;26:117. doi: 10.1186/s13059-025-03588-5 (PMC12060293; doi:10.1186/s13059-025-03588-5)
Supplement: Supplementary file 1 — Additional file 1: Supplementary figures. [file 13059_2025_3588_MOESM1_ESM.pdf]

Supplementary Figures

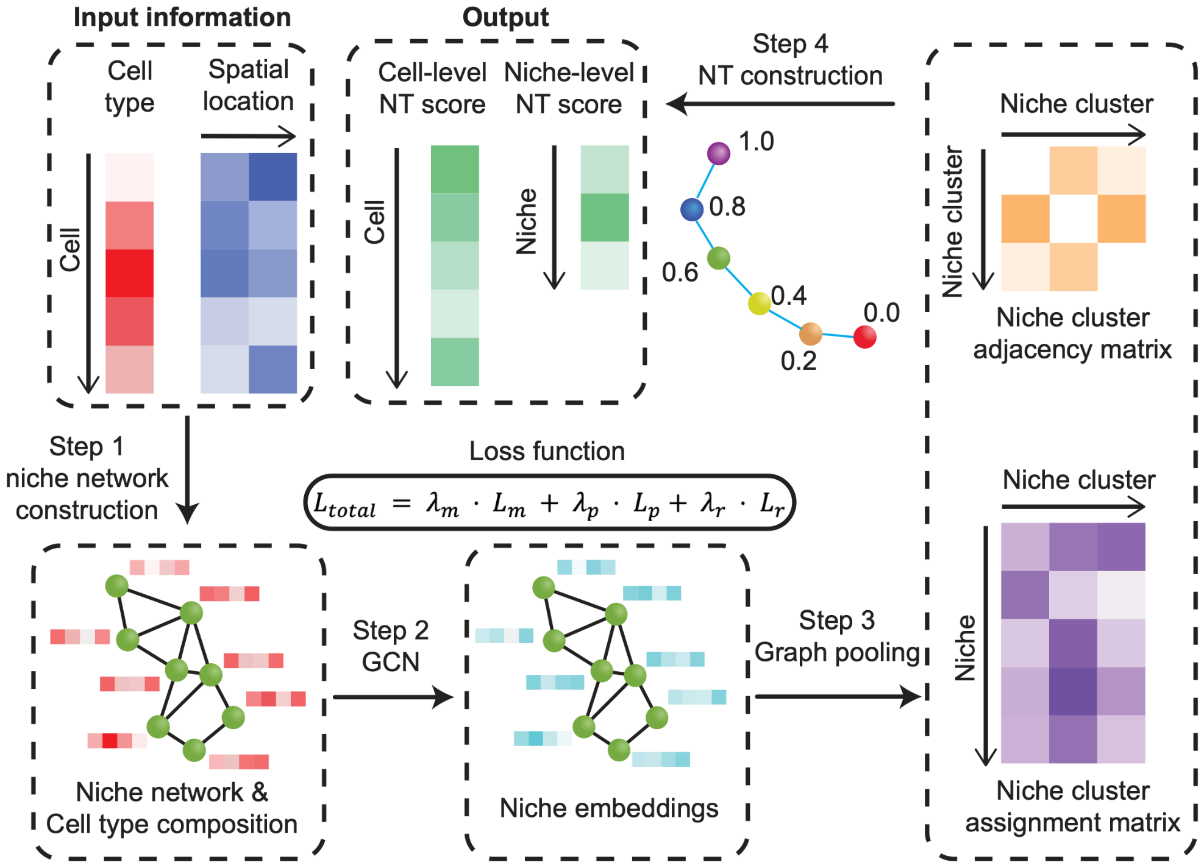

Fig. S1. A detailed schematic illustration of the ONTraC workflow. ONTraC takes cell-type annotation and spatial location information as input. The first step is to construct a niche network by using k-nearest neighbors in the physical space. Then, a two-layer graph convolution network (GCN) model is trained to encode data in a low-dimensional feature space. Next, a modified graph pooling approach is used to identify niche clusters and create a niche cluster network. Model training is done to minimize the loss function that considers three terms corresponding to modularity, purity, and regularization, respectively. The final step of niche trajectory construction is to order niche clusters so that total edge connectivity is optimized. Niche-level and cell-level NT scores are derived by projecting onto the resulting niche trajectory.

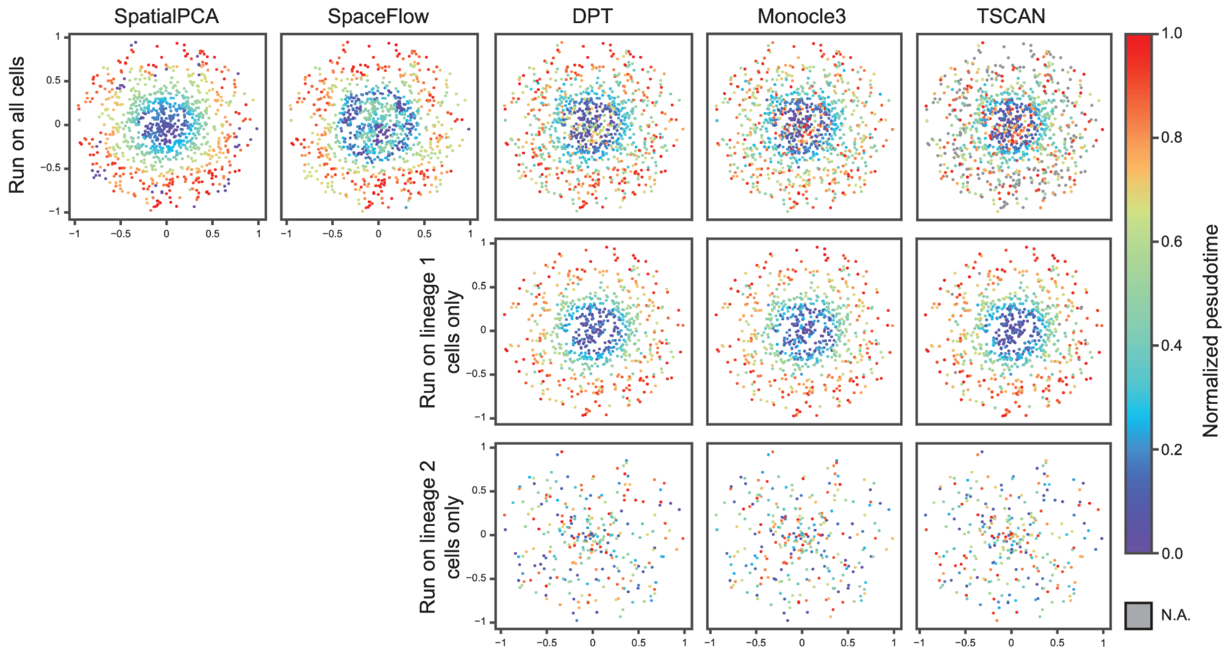

Fig. S2. Spatial patterns of pseudotime inferred from different methods for the simulated dataset. SpatialPCA and SpaceFlow were applied only to the whole dataset, whereas the other methods were applied to the whole dataset, lineage 1, and lineage 2, respectively. All pseudotime values were normalized to 0-1. Grey dots indicate the cells that received n.a. after pseudotime analysis.

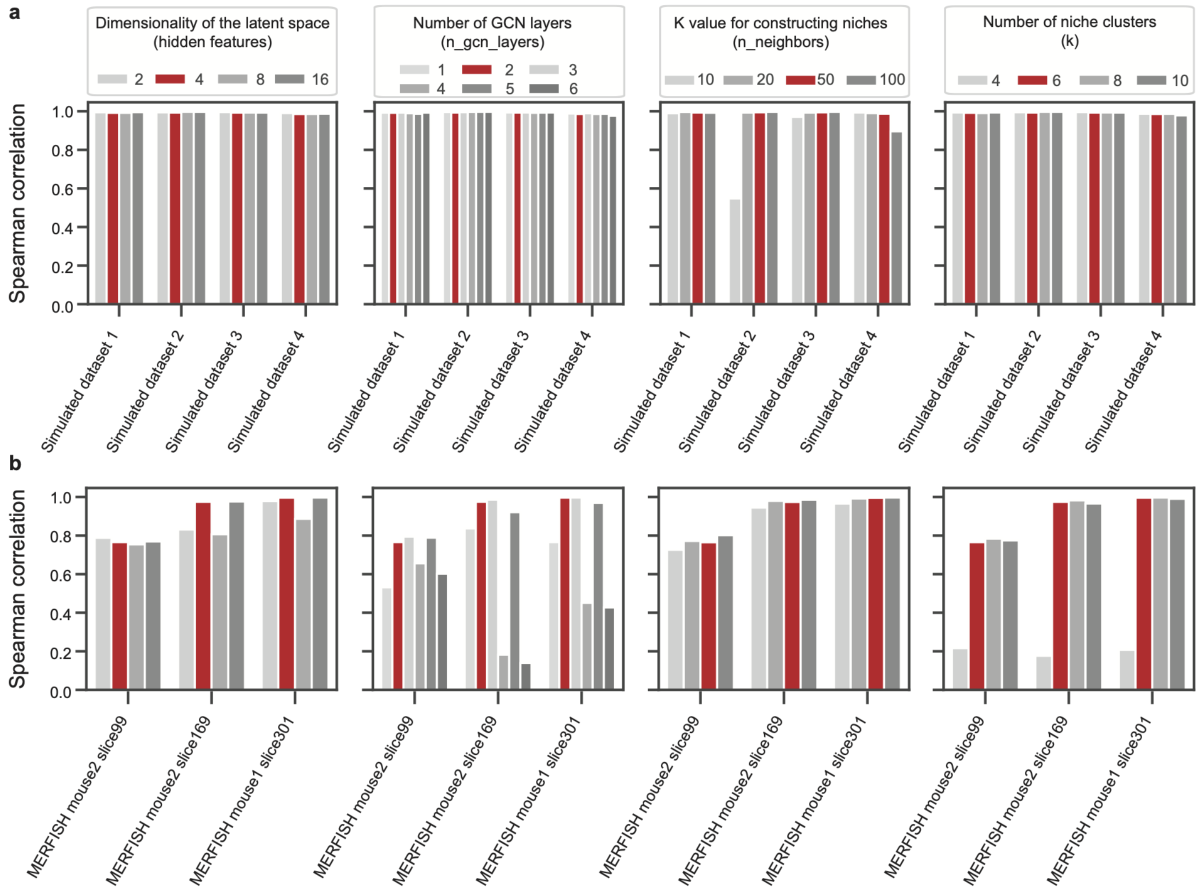

Fig. S3. Robustness analysis of ONTraC using different parameter settings. a, Simulated datasets. **b,** MERFISH datasets (the three representative samples shown in Fig. 1 were analyzed). Red bars indicate the default parameter values.

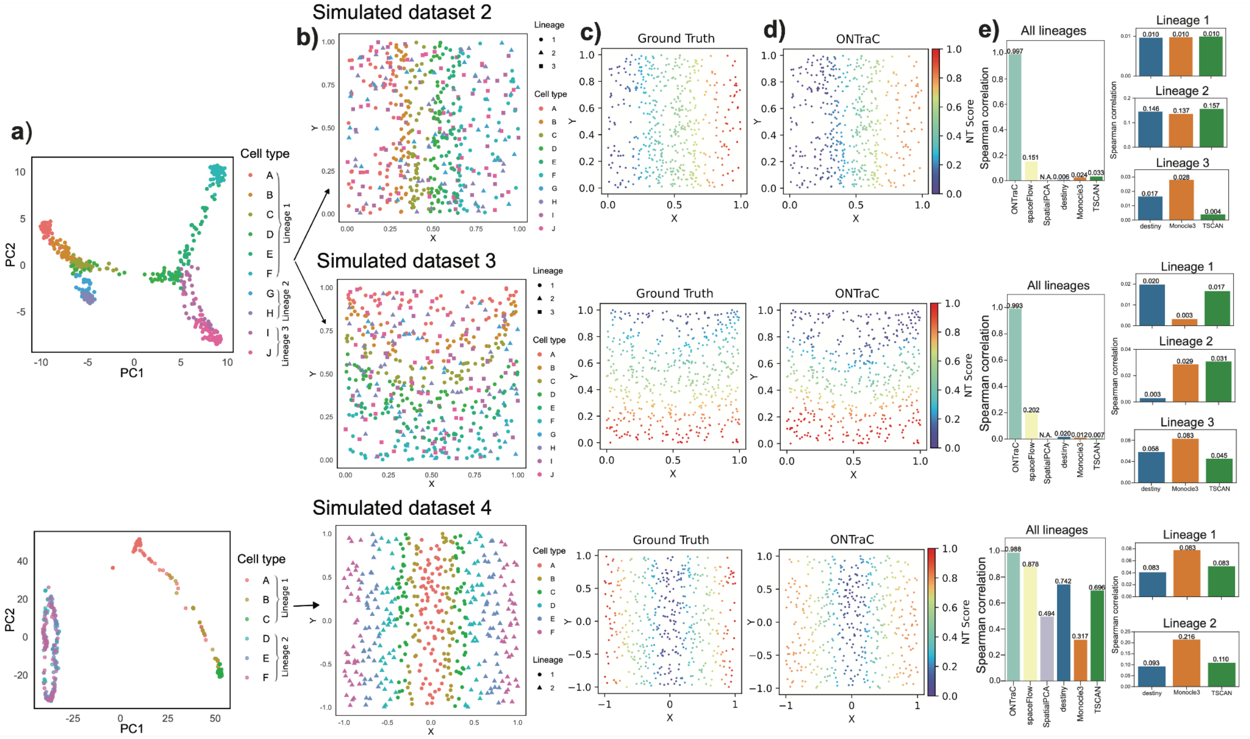

Fig. S4. Benchmark analysis using simulated datasets 2-4. a, PCA of the simulated gene expression pattern. **b,** Spatial distribution of cell types. **c,** Spatial distribution of ground truth. **d,** Spatial distribution of ONTraC predicted cell-level NT scores. **e,** Performance of different methods evaluated by the Spearman correlation between the ground truth and the ordering scores predicted by different methods. ONTraC, SpatialPCA and SpaceFlow were applied only to the whole dataset, whereas the other methods were applied both to the whole dataset and to each cell lineage separately.

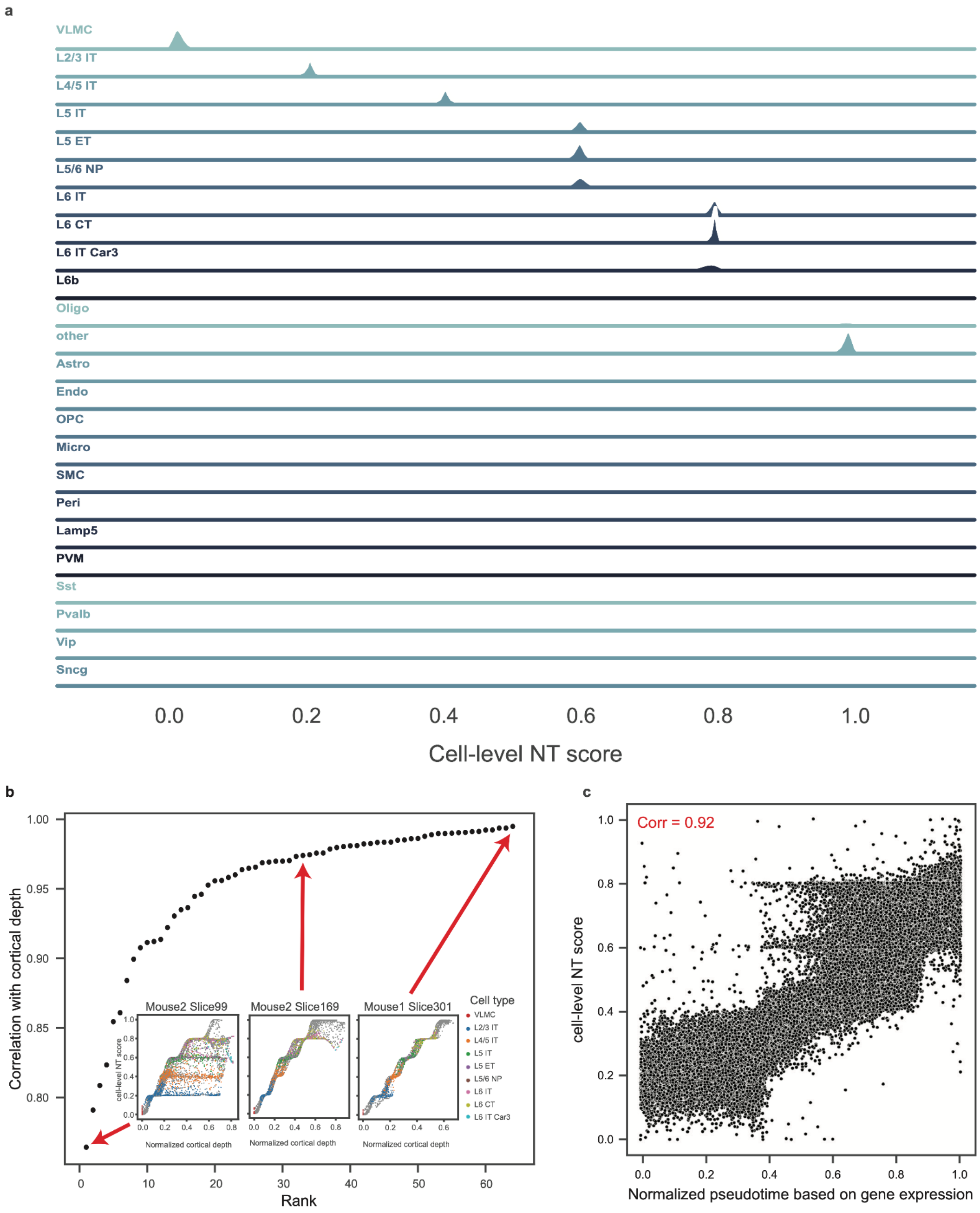

Fig. S5. ONTraC identifies niche trajectory on the mouse cortex MERFISH dataset. **a,** Cell type density along the reconstructed niche trajectory. **b,** Correlation between cell-level NT score and cortical depth for each tissue slice. Insets show scatterplot for the representative slices shown in **Fig. 1f-g. c,** Scatterplot showing the relationship between cell-level NT scores and normalized pseudotime on IT neurons.

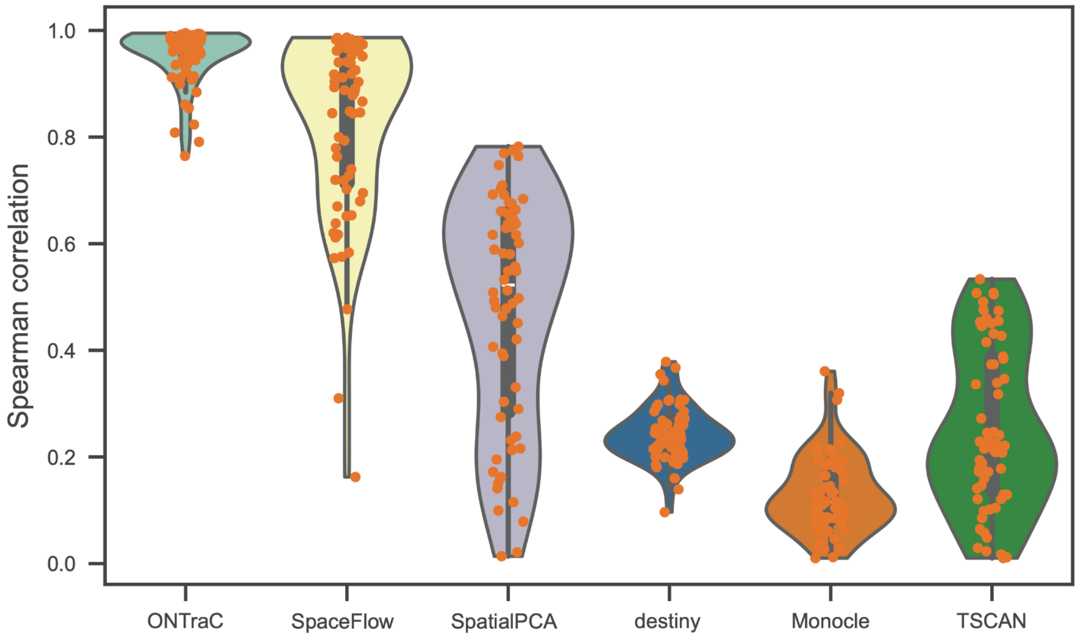

Fig. S6. Benchmark analysis using MERFISH dataset. Performance is evaluated by using the Spearman correlation between the cortical depth and the ordering scores predicted by different methods. SpaceFlow and SpatialPCA were run on each slice individually as they do not support multiple samples.

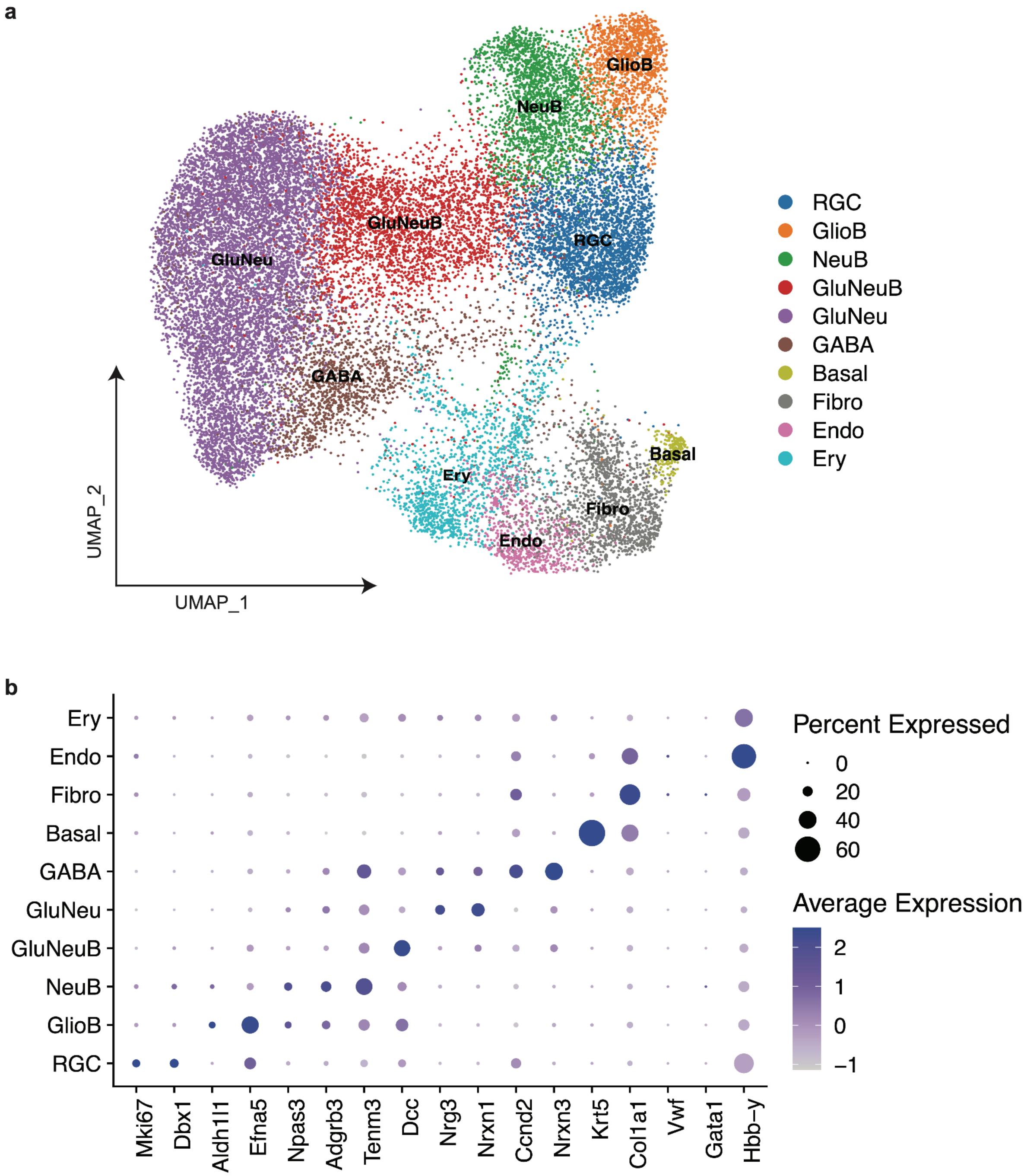

Fig. S7. Cell type annotation for in the mouse embryo dorsal midbrain stereo-seq dataset. a, Dimensionality reduction of gene expression data by UMAP along with annotated cell types. **b,** Cell-type specific marker gene expression patterns.

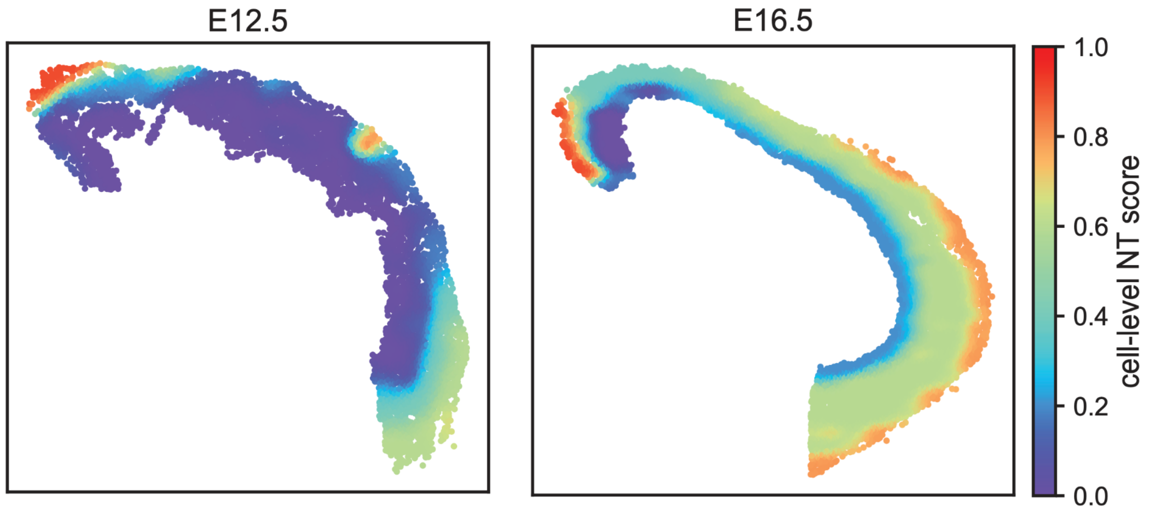

Fig. S8. Spatial distribution of cell-level NT scores in E12.5 and E16.5 dorsal midbrain.

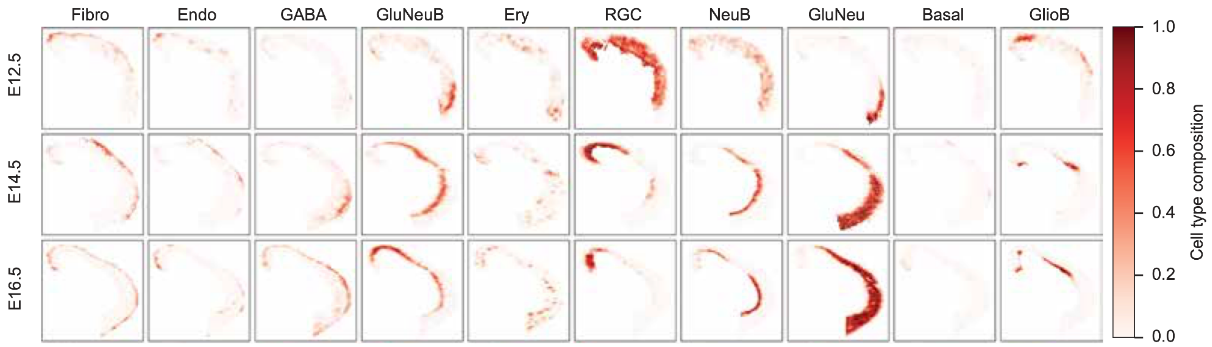

Fig. S9. Spatial distribution of different cell types in E12.5, E14.5, and E16.5 dorsal midbrain.

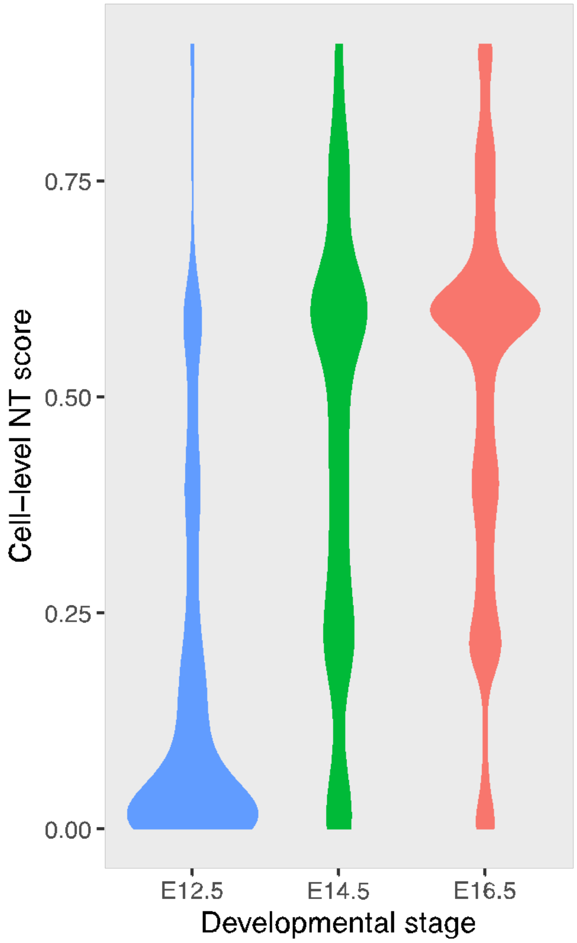

Fig. S10. Violin plots showing the overall distribution of cell-level NT scores in E12.5, E14.5, and E16.5 dorsal midbrain.

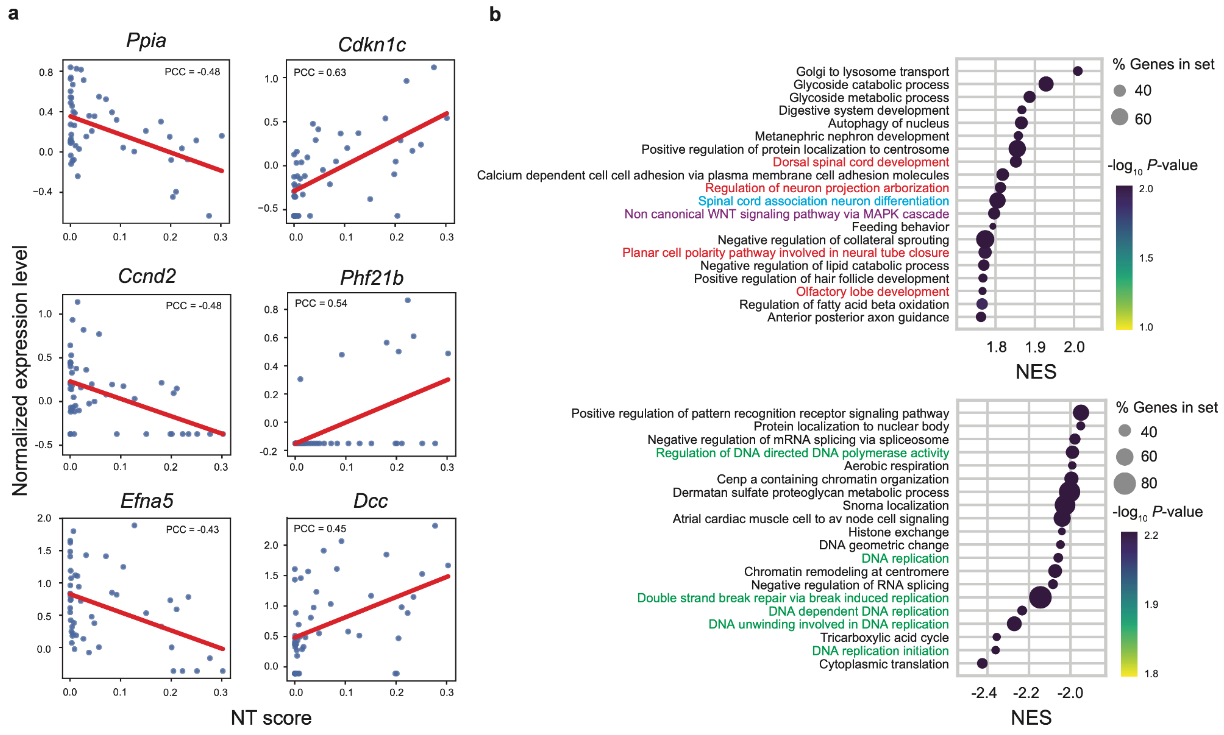

Fig. S11. Metacell-based correlation analysis between NT score and gene expression. a, Scatter plots show the represented genes relationship between gene expression and NT score. **b,** Top 20 positive/negative-associated gene ontology terms along niche trajectory. Neurogenesis-related terms are highlighted in blue (cell differentiation), red (nervous system development), and purple (WNT signaling pathway); DNA replication-related terms are highlighted in green.

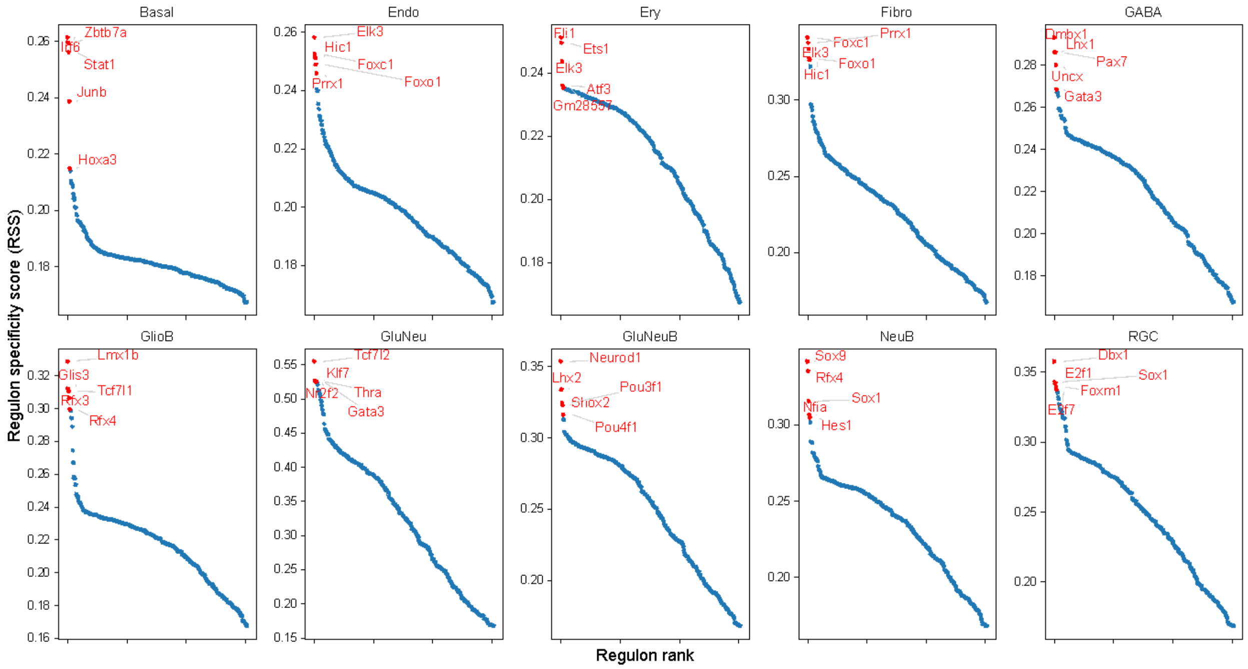

Fig. S12. Identifying cell-type specific regulators by ranking the regulon specificity scores (RSS). For each cell type, the top 5 regulons are highlighted.

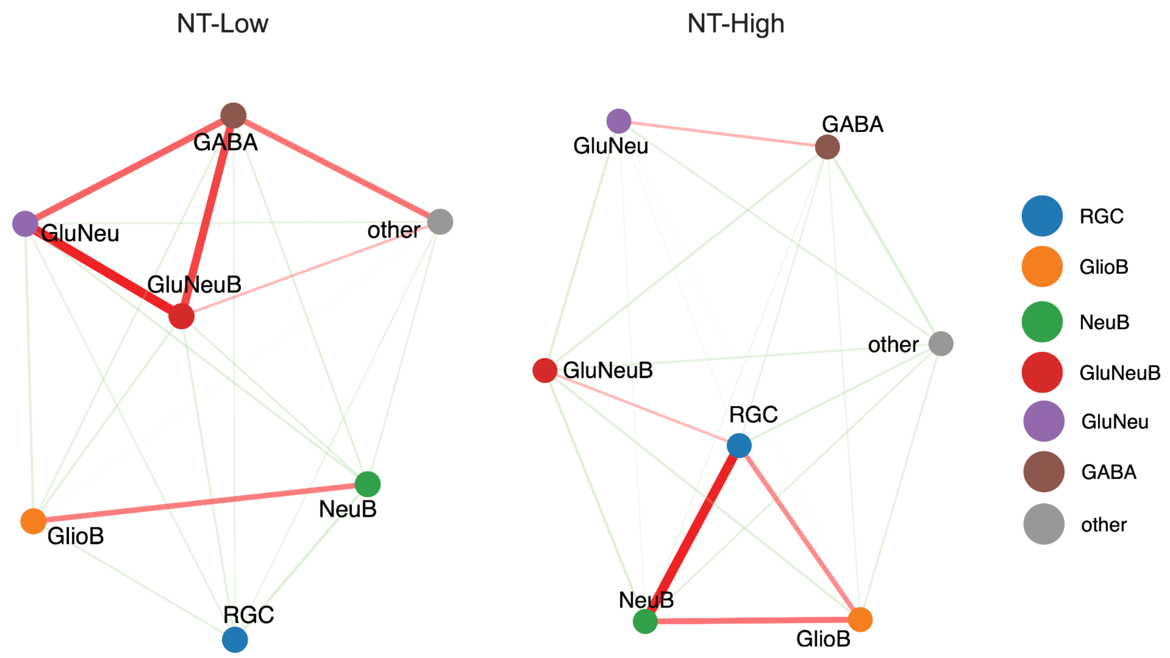

Fig. S13. Cell type proximity enrichment analysis for NT-Low and NT-High cells. Enriched or depleted interactions are depicted in red and green, respectively. Width of the edges indicates the strength of enrichment or depletion.

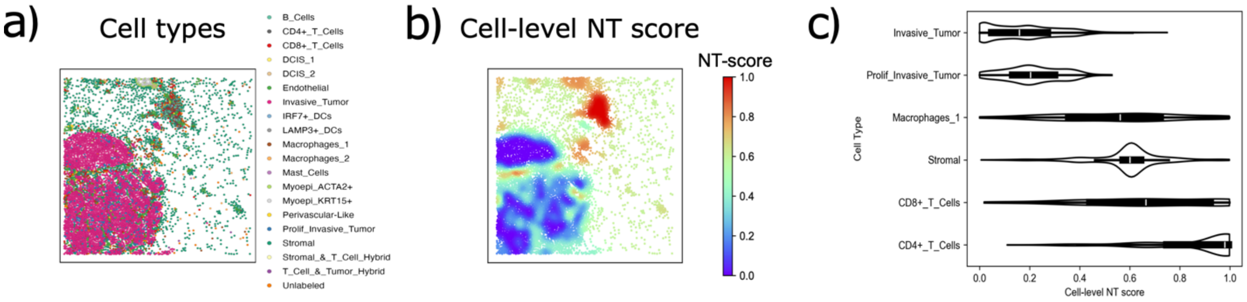

Fig. S14. ONTraC analysis of the Xenium breast cancer dataset. a, Spatial distribution of different cell types. **b,** Spatial distribution of the cell-level NT-scores. **c,** Cell type density along the reconstructed niche trajectory.

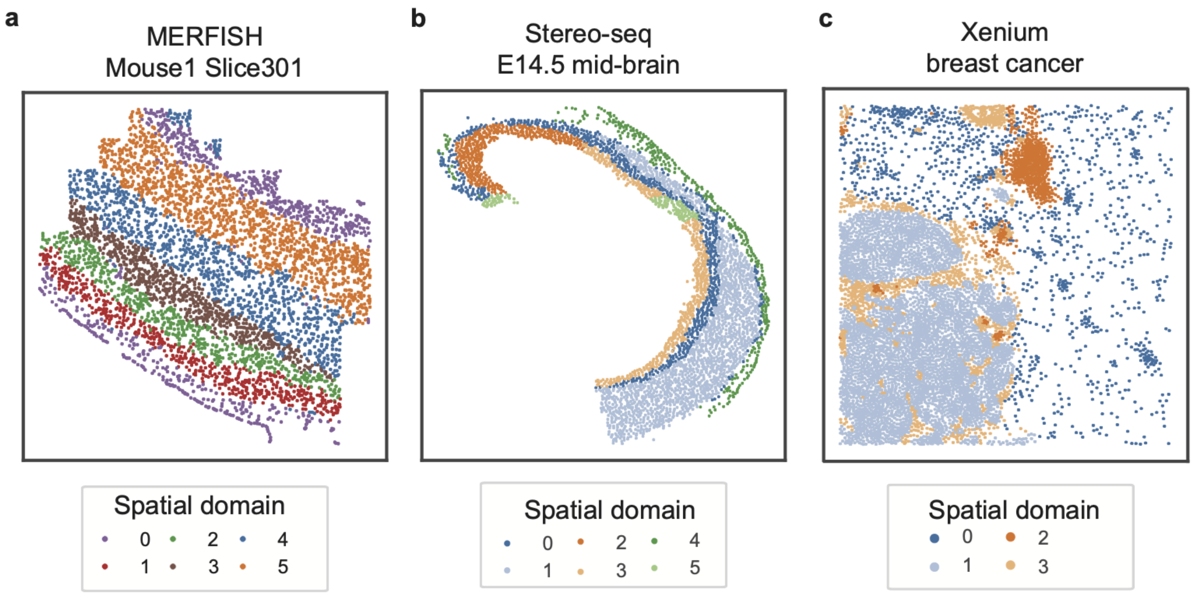

Fig. S15. Spatial domain analysis using GraphPCA. a, MERFISH mouse motor cortex data (mouse1 slice301 is shown as a representative example). **b,** Stereo-seq mouse embryo dorsal midbrain data. **c,** Xenium human breast cancer data.

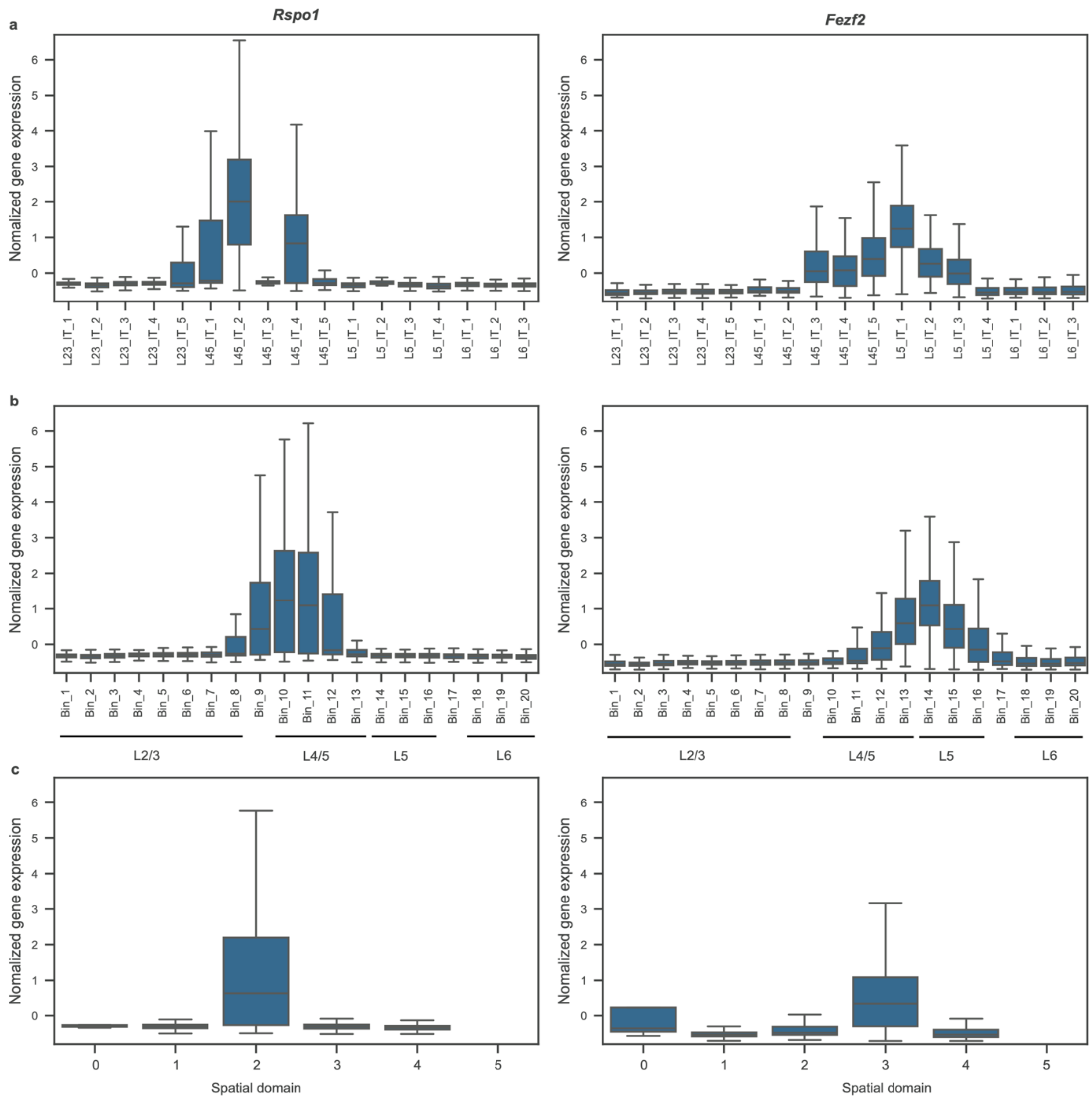

Fig. S16. ONTraC analysis reveals intra-layer heterogeneity of marker gene expression (using Rspo1 and Fezf2 as representative examples) in IT neurons. a, Marker gene expression level in IT neuron subtypes as annotated by the original paper (Zhang et al. 2021). **b,** Marker gene expression level in IT neuron subsets defined by binning NT-scores (corresponding cortical layers are marked below). A smooth change of gene expression can be observed along the change of NT scores. IT neurons in Bin 9 are a mixture of L2/3 and L4/5 IT neurons. IT neurons in Bin 17 are a mixture of L5 and L6 IT neurons. **c,** Marker gene expression level in IT neurons associated with GraphPCA predicted spatial domains. The three representative samples shown in Fig. 1f-g were used in this analysis.

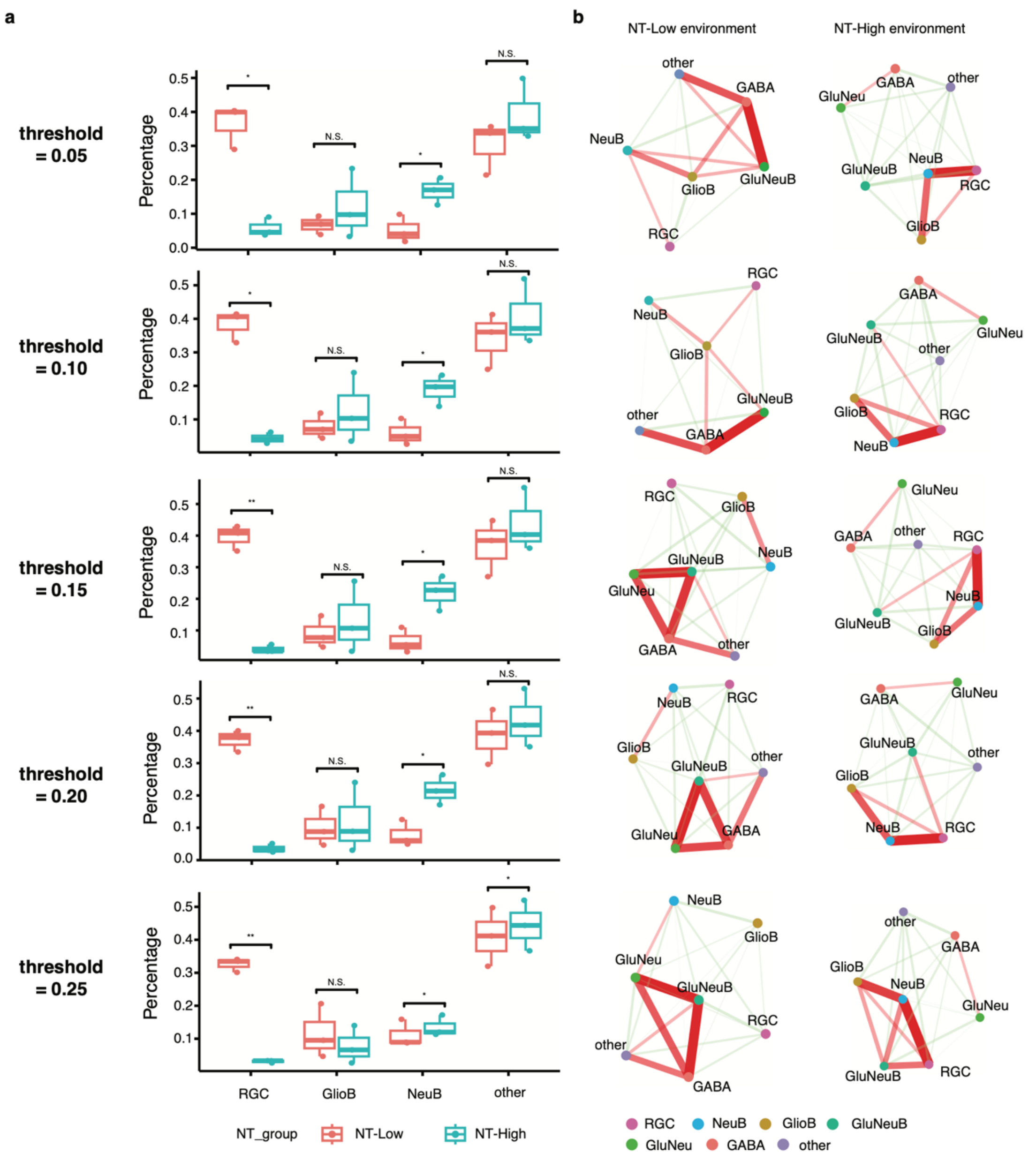

Fig. S17. Comparative analysis of NT-High vs NT-Low cells using different threshold values. a, Box plots showing the cell-type composition of putative offspring cells of E14.5 NT-Low and NT-High RGCs. NT-High RGCs show lower self-renewal and higher differentiation potential compared to NT-Low RGCs under all thresholds. **b,** Cell type proximity enrichment analysis. NT-High RGCs show higher proximity to NeuB and GlioB under all thresholds. Enriched or depleted interactions are depicted in red and green, respectively. Width of the edges indicates the strength of enrichment or depletion.
